# Mycobacterial Mutants with Defective Control of Phagosomal Acidification

**DOI:** 10.1371/journal.ppat.0010033

**Published:** 2005-11-25

**Authors:** Graham R Stewart, Janisha Patel, Brian D Robertson, Aaron Rae, Douglas B Young

**Affiliations:** 1 Department of Infectious Diseases and Microbiology, Centre for Molecular Microbiology and Infection, Imperial College London, London, United Kingdom; 2 School of Biomedical and Molecular Sciences, University of Surrey, Surrey, United Kingdom; University of Washington, United States of America

## Abstract

The pathogenesis of mycobacterial infection is associated with an ability to interfere with maturation of the phagosomal compartment after ingestion by macrophages. Identification of the mycobacterial components that contribute to this phenomenon will allow rational design of novel approaches to the treatment and prevention of tuberculosis. Microarray-based screening of a transposon library was used to identify mutations that influence the fate of *Mycobacterium bovis* bacille Calmette-Guérin (BCG) following uptake by macrophages. A screen based on bacterial survival during a 3-d infection highlighted genes previously implicated in growth of *Mycobacterium tuberculosis* in macrophages and in mice, together with a number of other virulence genes including a locus encoding virulence-associated membrane proteins and a series of transporter molecules. A second screen based on separation of acidified and non-acidified phagosomes by flow cytometry identified genes involved in mycobacterial control of early acidification. This included the KefB potassium/proton antiport. Mutants unable to control early acidification were significantly attenuated for growth during 6-d infections of macrophages. Early acidification of the phagosome is associated with reduced survival of BCG in macrophages. A strong correlation exists between genes required for intracellular survival of BCG and those required for growth of *M. tuberculosis* in mice. In contrast, very little correlation exists between genes required for intracellular survival of BCG and those that are up-regulated during intracellular adaptation of *M. tuberculosis*. This study has identified targets for interventions to promote immune clearance of tuberculosis infection. The screening technologies demonstrated in this study will be useful to the study of pathogenesis in many other intracellular microorganisms.

## Synopsis

The pathogenesis of *Mycobacterium tuberculosis* relies on an ability to survive inside host macrophages. Macrophages kill most other bacteria by engulfment into an intracellular compartment called a phagosome, which quickly matures to an acidic, hydrolytic organelle, resulting in bacterial death. *M. tuberculosis* and the related vaccine strain *M. bovis* bacille Calmette-Guérin (BCG) possess the ability to stop phagosome maturation and thus avoid its microbicidal properties. In this study, the researchers screened a library of mutant BCG bacteria to identify the bacterial genes responsible for preventing phagosome acidification. The predicted products of these genes span many different functional groups, but tend to be associated with the outside of the cell or secreted to the extracellular milieu. The researchers also demonstrated that mutant mycobacteria whose phagosomes acidify are unable to replicate in macrophages. This study identifies targets for new vaccines against tuberculosis.

## Introduction

*Mycobacterium tuberculosis* presents a major challenge to global health, claiming around two million lives every year. The success of this pernicious pathogen centres around an ability to avoid destruction by the immune response throughout the course of an infection that may last the lifetime of the host. To achieve this, the bacilli have adapted to survive and replicate inside the cells of the host, principally within the very immune cell that is designed to destroy invading microorganisms, the macrophage. Multiple factors are involved in intracellular survival [1]. A degree of inherent resistance to killing is conferred by the mycobacterial cell wall, which presents a relatively impervious physical barrier to the hydrolytic enzymes encountered within macrophages, and by mycobacterial enzymes that detoxify reactive radicals. To avoid activation of macrophages by T cells, *M. tuberculosis* interferes with dendritic cell function [2,3], antigen presentation [4,5], and cytokine signalling by infected cells [6]. But perhaps the most striking feature of pathogenic mycobacteria is an ability to arrest the normal process of phagosome maturation, allowing survival in a non-acidified intracellular compartment [1].

Normal phagosomes undergo a process of maturation that is driven by continual fusion and fission events with other endosomal compartments [7], resulting in acquisition of late endosome/lysosome-associated proteins via a biosynthetic trafficking pathway from the trans-Golgi network (TGN) [1,8]. Early phagosomes share many properties with early endosomes and are characterised by markers such as the small GTPase Rab5 [9] and its effector, early endosomal antigen 1 [10]. Within minutes of formation, however, phagosomes begin to accumulate other markers corresponding to those of late endosomes. For example, while early endosomal antigen 1 and Rab5 are lost, there is a gradual accumulation of another GTPase, Rab7 [11], which is involved in fusion of late endosomes and lysosomes [12]. Concurrent with these changes in membrane markers and fusogenicity of the phagosome are changes in the physiology of the phagosomal lumen, including its acidification from a pH of 5.5 in late phagosomes to 4.5 in phagolysosomes. This occurs by the accumulation of the vacuolar proton ATPase (V-ATPase). The V-ATPase may be sourced from the TGN [13] and from fusion with other compartments, particularly V-ATPase-rich lysosomes.

Slow-growing mycobacteria—such as *M. tuberculosis, M. avium,* and the attenuated bacille Calmette-Guérin (BCG) vaccine strain of *M. bovis*—interfere with this process, occupying an atypical phagosome arrested at the early phagosome stage [14–16]. The mycobacterial phagosome retains Rab5 [17–19] and maintains fusogenicity with early endosomes in the rapid recycling pathway, as evidenced by its access to transferrin [20,21]. However, it does not fuse with late endosomes/lysosomes or accumulate associated markers. In addition, it does not accumulate V-ATPase, and it fails to acidify, remaining at pH 6.3 [22]. Phagosomal arrest is specific to the live organism; phagosomes occupied by killed mycobacteria rapidly acidify and fuse with lysosomes [1].

Elucidation of the molecular mechanisms by which mycobacteria arrest phagosome maturation may uncover novel intervention targets for disease control. It is clear that the surface properties of the organism have an important influence on this process, and several mycobacterial lipids and glycolipids have been implicated in altered phagosome biogenesis [13,23–25]. The involvement of mycobacterial viability in efficient phagosomal arrest suggests that such structural effector mechanisms are complemented by active interference in macrophage cell biology. Recent reports have described a serine-threonine kinase, PknG, of pathogenic mycobacteria that could modify host proteins involved in control of intracellular trafficking pathways, for example [26], and exclusion of phosphatidylinositol-3-phosphate from phagosomes containing live mycobacteria [27]. In a study based on screening of mycobacterial mutants, Pethe and colleagues identified multiple genes required for phagosomal arrest [28], including those involved in biosynthesis of surface lipids, transport of possible effector molecules, and production of isoprenoid compounds. Taken together, these studies suggest that phagosomal arrest represents a complex multigenic phenotype.

The combination of whole-genome transposon mutagenesis with microarray-based, high-throughput analysis presents an attractive approach to study such multicomponent systems. In two seminal papers, Sassetti and colleagues have used this approach to provide an overview of mycobacterial genes required for optimal growth in laboratory culture and in a mouse infection model [29,30]. The aim of the present study was to use an analogous approach to investigate phagosomal arrest. We analysed a transposon library in *M. bovis* BCG using biological screens that were designed to identify mutants that were defective (a) in intracellular survival, and (b) in prevention of early phagosomal acidification. We anticipated that this would allow us to identify novel individual genes involved in each of these processes and, by comparing the two datasets, to determine whether the two phenotypes are functionally linked.

## Results

### Transposon Screen by Microarray

**Figure 1:**
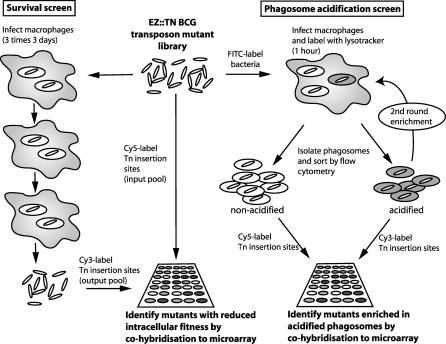
High-Throughput Screening for Mycobacterial Mutants with Reduced Survival in Macrophage Culture, or Defective Ability to Arrest Phagosome Acidification For the survival screen, the BCG transposon library was selected through three rounds of macrophage culture (72 h each) and survival of mutants assessed by fluorescent labelling of transposon insertion sites in input and output mutant pools and competitive hybridisation to a microarray. To screen for mutants unable to inhibit phagosome acidification, the transposon mutants were FITC-labelled and used to infect macrophages in the presence of LysoTracker for 1 h. Organelles were prepared and acidic phagosomes sorted by flow cytometry. Mutants in the acidic phagosomes were grown in broth and used for a second round of selection. The identity and abundance of mutants recovered from acidic and non-acidic phagosomes were compared by fluorescent labelling of transposon insertion sites and hybridisation to a microarray.

A derivative of the Epicentre EZ::TN transposon carrying a hygromycin-resistance determinant (EZ::TN*hyg* ) was used to generate a transposon library in *M. bovis* BCG. Sequencing of insertion sites within specific genes revealed 9-bp direct repeats flanking the transposon, thus indicating genuine Tn5 transposition [31]. Detailed analysis of 30 insertions revealed some site preference, with position 1 of the direct repeat biased to G and position 9 to C in greater than 60% of events (Table S1). In GC-rich genomes such as BCG and *M. tuberculosis,* this bias may represent an advantage that makes EZ:TN a useful addition to *Himar1* [29,30] and IS*1086*-based transposons [32–34]. Pooled mutants were analysed by hybridisation of fluorescently labelled transposon insertion sites to whole-genome microarrays. We evaluated the reproducibility of the transposon screen by microarray (TSM) labelling and hybridisation process by comparing fluorescent intensities of hybridising spots derived from two independent labelling experiments performed on the same 2,500-clone library (Figure S1A). A highly significant correlation between the different experiments (*r* ^2^ = 0.9289) demonstrated the reproducibility of the method. The microarray readout of insertion site locations within the transposon pool provides an ideal method for examining the genome-wide distribution of transposition, and showed that the EZ::TN*hyg* transposon was generally well distributed throughout the entire genome (Figure S1B). We have not observed any occurrences of multiple transposon insertions within a single cell. We next examined the effect on the pool of exposure to biological selections based on intracellular survival in macrophages and on phagosomal acidification (Figure 1).

### Transposon Screen to Quantify Intracellular Fitness

**Figure 2:**
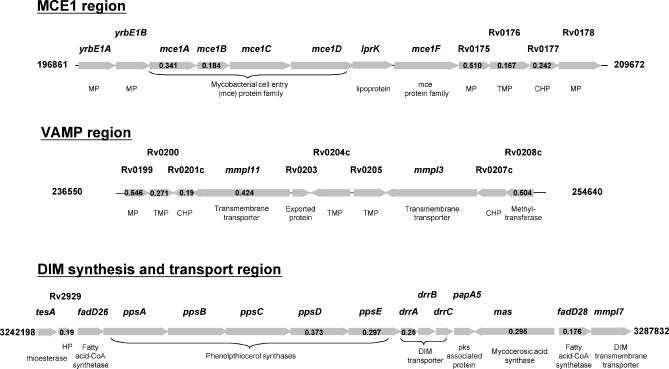
Gene Regions with Low Intracellular Fitness Ratios The identification of known virulence regions such as the *mce1* operon and the DIM synthesis and transport region, validate the TSM intracellular fitness assay. The strategy also identified virulence regions such as *VAMP*. Region coordinates derived from *M. tuberculosis* H37Rv are shown. FRs (calculated by dividing output signal intensity by input signal intensity*)* are shown inside horizontal grey arrows representing each open reading frame (open reading frames without ratios did not generate hybridisation signals). CHP, conserved hypothetical protein; HP, hypothetical protein; MP, membrane protein; TMP, transmembrane protein.

TSM was used to identify insertions associated with decreased intracellular fitness by comparing transposon pools before and after three rounds of 72-h culture in macrophages. Results of four replicate screens from each of two independent experiments with the same input pool were combined to generate a mean fitness ratio (FR) for more than 1,500 genes, representing approximately 60% of approximately 2,600 non-essential genes in the BCG genome; this information is provided together with an ordered list of the top 100 attenuating insertions in Tables S2 and S3. The 20 most strongly attenuating insertions are shown in Table 1. As the BCG genome has yet to be annotated and most existing datasets relate to nomenclature based on the *M. tuberculosis* H37Rv sequence, we have used “Rv” numbers to identify genes throughout this study. We screened this list initially for the presence of genes previously shown to be required for survival of mycobacteria in macrophages. Insertions with a high fitness cost include those in a known virulence locus involved in synthesis and transport of phthiocerol dimycocerosate (DIM), a complex lipid component of the mycobacterial cell wall [32,33], and in the *mycobacterial cell entry 1 (mce1)* region (Rv0169–Rv0178), which has been implicated in mycobacterial entry to host cells [35–37] and is required for virulence in a mouse model (Figure 2) [30]. Reduced fitness (FR < 0.5; false discovery rate [FDR] = 1%) was also associated with a series of insertions in the pathway for conversion of isocitrate to glucose (Table S3)—isocitrate lyase (*icl,* Rv0467), PEP carboxykinase (*pcka,* Rv0211) and fructose-1,6-bisphosphatase (Rv1099c)—consistent with previous proposals that mycobacteria rely predominantly on lipid substrates for intracellular metabolism [38]. The prominence of previously described attenuating mutations provides a validation of the effectiveness of the intracellular fitness screen.

**Table 1:**
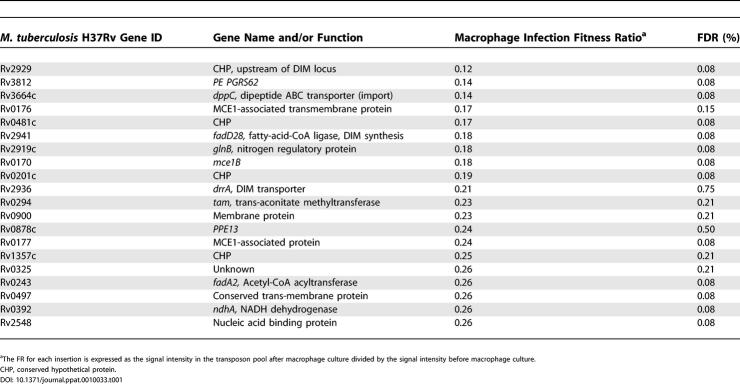
Mutants That Incurred the Highest Fitness Cost in Macrophage Culture

We next searched the data for other operons or regions that contained multiple transposon hits with low intracellular fitness scores. The four spots representing Rv0199–Rv0202c had significantly reduced fitness ratios (FR = 0.19–0.55; FDR < 2%), with Rv0200 and Rv0201c amongst the most highly attenuated mutants (Tables 1, S2, and S3). Insertional mutation of several genes in the region from Rv0199 through Rv0208c has been reported to confer a significant growth defect in mice [30], and Rv0204c was picked out as required for optimal in vivo growth in a previous signature-tagged mutagenesis screen [32]. Due to the abundance of genes encoding membrane-associated proteins in this region, we have termed it the virulence-associated membrane protein region, or VAMP (Figure 2). The region is one example of several that highlight a central requirement for export of macromolecules during mycobacterial growth in macrophages. Rv0199 and Rv0200 encode transmembrane proteins related to Rv1972/1973 and Rv0177/0178 present in the *mce3* and *mce1* loci, respectively. Rv0202c and Rv0206c encode members of the RND superfamily, referred to as MmpL11 and MmpL3 and predicted to act as lipid transporters. Rv0204c and Rv0205 encode conserved transmembrane proteins, predicted in the latter case to function as a permease. Rv0203 encodes an exported protein, the secretion signal of which contains a characteristic motif with adjacent twin arginine residues, that is associated with proteins that are exported by the Sec-independent Tat pathway [39]. Tat-exported proteins have been implicated in the pathogenesis of several bacteria; homologues of the *tatA* and *tatC* genes are present in the genome of *M. tuberculosis,* but the function of this secretion pathway has yet to be investigated in mycobacteria. Further examples of attenuating lesions associated with predicted secreted proteins include insertions in adjacent genes encoding members of the ESAT-6 family (Rv3444c and Rv3445c) [40,41]. The insertion with the second highest fitness costs mapped to Rv3812, a gene encoding a member of the PE PGRS family of predicted cell surface proteins that has been linked to chronic infection by *M. marinum* [42].

Small molecule transporters were also prominent amongst the most attenuating mutations. These include a dipeptide transporter (*dppC,* Rv3664c) that also displays a marked phenotype in the mouse virulence model [30], two genes involved in uptake of asparagine (*ans,* Rv2127 and *aroP2,* Rv0346c), and transporters for sugars (*sugA,* Rv1236), ribonucleotides (*mkl,* Rv0655), and sialic acid (*nanT,* Rv3294). Other transporters associated with virulence impairments are predicted to mediate uptake or export of cations (*ctpG,* Rv1992c and *mgtE,* Rv0362) and drugs (Rv1272c and Rv1410c).

### Transposon Screen for Mycobacterial Genes Involved in Control of Phagosome Acidification

**Figure 3:**
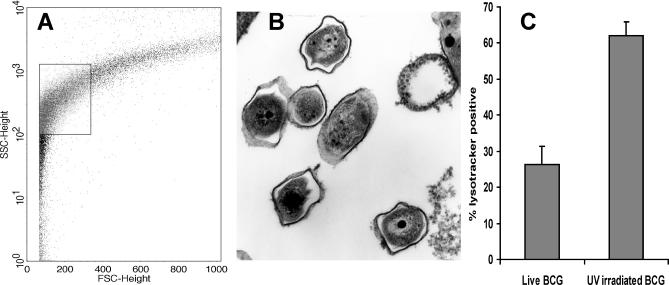
Fluorescence-Activated Cell Sorting of Mycobacterial Phagosomes (A) Forward and side scatter plot of organelle preparation from BCG-infected J774 macrophages. Gated area denotes the population which includes mycobacterial phagosomes, based on the signal from FITC-labelled mycobacteria. (B) Using fluorescently labelled mycobacteria, phagosomes were sorted into fixative for confirmation of identity and integrity. Fluorescent events in the gated population predominantly consisted of mycobacterial phagosomes with bacteria surrounded by intact membranes. (C) Flow cytometric analysis of acidification of mycobacterial phagosomes using the acidotropic dye LysoTracker DND99. Significantly more UV-killed BCG localised to acid compartments at 1 h post-infection (*p* < 0.01).

To screen for mutations affecting phagosomal acidification, we developed flow cytometry techniques for analysis and sorting of these organelles. Macrophages infected for 1 h with fluorescence-tagged BCG were lysed and, after disruption of the cytoskeleton and removal of nuclei, organelles were analysed by flow cytometry. The fluorescent tag, combined with forward- and side-scatter profiles, provided a marker to identify the mycobacteria-containing compartments (Figure 3A). To verify that these fluorescent events represented intact mycobacterial phagosomes, we sorted the gated particle population into fixative and confirmed by electron microscopy that it contained large numbers of mycobacteria enclosed by unbroken membranes (Figure 3B). Phagosomes were further sorted into acidified and non-acidified compartments using the fluorescent dye LysoTracker DND99, which accumulates in acidic compartments and has previously been successfully exploited to monitor acidification of the mycobacterial phagosome by confocal microscopy [18]. We used flow cytometry to compare the proportion of LysoTracker -positive phagosomes obtained after infection with live and dead BCG. Approximately 26% (standard deviation [SD] = 8.6%) of live BCG phagosomes were LysoTracker-positive compared to 62% (SD = 6.7%) of phagosomes containing dead bacteria (Figure 3C). This is consistent with previous reports that live mycobacteria inhibit acidification of the phagosome and validated the use of flow cytometric detection of LysoTracker accumulation in phagosomes as a method to differentiate bacteria by their ability to arrest acidification**.**

We infected macrophages with a pool of 2,500 BCG EZ::TN*hyg* mutants and used flow cytometry to select the LysoTracker-positive phagosome population that we anticipated would be enriched for mutants unable to arrest acidification. This was confirmed by the observation that the proportion of acidified phagosomes increased from 35% during the first flow-cytometric selection to 51% in a second, subsequent round of enrichment. A final pool of mutants from the two rounds of enrichment was compared to the pool of mutants present in the LysoTracker-negative phagosomes by TSM (see Figure 1). Fold enrichment in the “acidified” pool was calculated as the ratio of signal intensity in the LysoTracker-positive pool over signal intensity in the LysoTracker-negative pool. Spots with a high fold enrichment indicated genes in or near which a transposon insertion conferred the greatest reduction in ability to control phagosome acidification.

Results from four replicate screens from two independent experiments were combined to generate the mean fold enrichment. Data were considered statistically significant when the FDR was calculated to be less than 5%. Insertion in 43 genetic regions had a significant effect on phagosome acidification (Table S4); the top ten insertions (FDR < 2%) are shown in Table 2. Sequence analysis showed that eight of the ten insertions had occurred within the gene indicated by the microarray, one was located just within the 5′ end of an adjacent gene, and one could not be amplified and sequenced (Table 2). We thus consider that the hybridisation pattern revealed by the TSM technique relates well to the actual mutagenised genes.

**Table 2:**
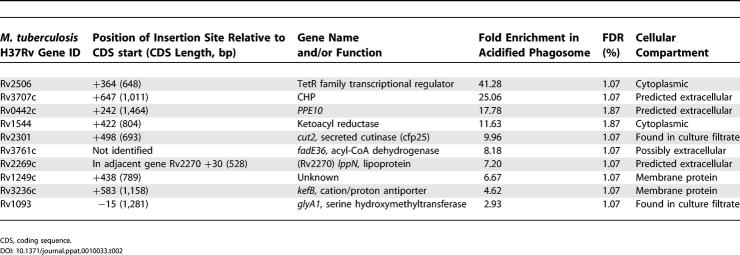
The Ten Mutants Most Significantly Enriched in Acidified Phagosomes

To confirm by an alternative technique that the screen had correctly identified mutations with a reduced ability to inhibit phagosome acidification, we isolated four transposon mutants from the acidification-enriched pool (Tn::Rv3707c, Tn::*PPE10,* Tn::*cut2,* and Tn::*glyA1*). Macrophages infected with the individual mutants were examined by confocal microscopy. After a 1-h infection, all four mutant strains co-localised with LysoTracker at a significantly increased frequency compared to wild-type (Table 3), thus further confirming the validity of the flow cytometric selection procedure.

**Table 3:**
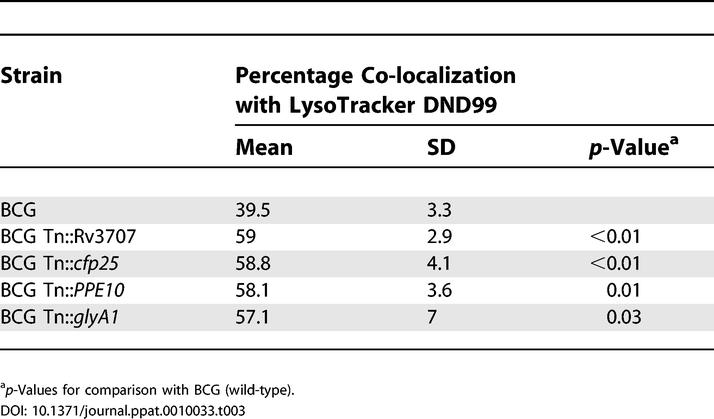
Confocal Microscopy Analysis of FACS-Selected Mutants Confirms Increased Localisation in Acidic Phagosomes

The top ten genes (see Table 2) span a range of predicted functional classes, but it is notable that eight share the property of encoding proteins associated either with the outside of the cell or with the extracellular milieu. These include three predicted surface proteins of unknown function: a member of the PPE family (PPE10), a lipoprotein (LppN), and a conserved hypothetical protein (Rv3707c). The list includes two secreted enzymes: *cut2,* encoding a member of a family of serine esterases with significant similarity to fungal cutinases, and *glyA1,* a serine hydroxymethyltransferase that is found in the culture filtrate of *M. tuberculosis* [43]. Insertion in *kefB* provides the most obvious connection between gene function and altered phagosomal pH. KefB is a potassium efflux system that functions via potassium/proton antiport [44,45] and in *Escherichia coli* acts to acidify the bacterial cytoplasm to protect the cell from electrophile toxicity [46,47]. For a bacterium in the phagosome, the release of potassium from the cytoplasm via KefB would be compensated by uptake of protons from the phagosomal lumen, resulting in an increase in lumenal pH. A mycobacterial mutant lacking *kefB* would thus have a reduced ability to actively alter cation proton balance in its own cytoplasm and consequently also in the phagosomal lumen.

### Intracellular Fitness of Mutants Whose Phagosomes Acidify

**Figure 4:**
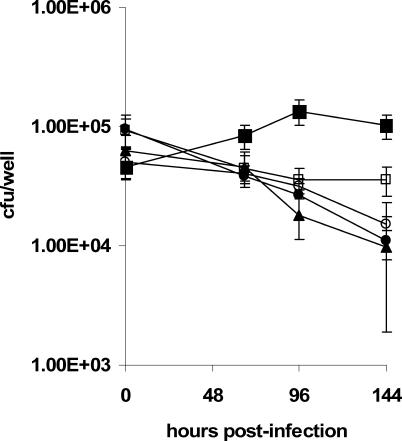
Reduced Intracellular Fitness of Mutants That Failed to Arrest Phagosome Acidification Mutants that failed to inhibit the acidification of their phagosomes were assessed for survival over 6 d in J774 macrophages. BCGPasteur (solid squares), Tn::Rv3707c (open squares), Tn::*PPE10* (closed circles), Tn::*cut2* (solid triangles), Tn::*glyA1* (open circles). Error bars indicate ± 1 SD.

While each of the biological screens confirmed and expanded our understanding of the biology of mycobacteria-macrophage interactions, we were surprised to observe little overlap between the two datasets. Only two (Rv2506 and Rv3057) of the 43 insertions identified on the basis of their failure to control phagosome acidification (Table S4) were amongst the top 100 insertions that compromised intracellular growth over the 72-h infection (see Table S3). Indeed, the overall distribution of intracellular fitness values for the acidification-enriched mutants was not significantly different from that of the whole transposon library (*p* < 0.38, t-test) (Figure S2). In light of this result, we examined the intracellular fitness of the individual Tn::Rv3707c, Tn::*PPE10,* Tn::*cut2,* and Tn::*glyA1* “acid phagosome” mutants during 6-d (144-h) macrophage infections. All four mutants were significantly attenuated (*p* < 0.05) for survival, but this was most pronounced after 4 d and 6 d of infection (Figure 4).

## Discussion

The EZ::TN system provides a tool that is complementary to IS*1096*-based transposons and *Himar1* [29,30,32–34] for generation of transposon libraries in slow-growing mycobacteria. Application of a simple PCR amplification allows it to be used in combination with high-thoughput microarray screening, bypassing the need for construction of a customised transcription-based readout required for related systems [29,48]. Approaches based on TSM are attractive in providing a global overview of genes involved in a complex biological system, particularly in the search for mutations that are negatively selected due to loss of biological function or viability. Drawbacks of such approaches can include a lack of precision—depending on the precise location of an insertion, it may generate a signal from adjacent genes, for example—and aberrant behaviour displayed by individual clones when present as a member of a pool of organisms. It is therefore often necessary to confirm results from such screens by targeted analysis of individual mutants. The two screens described in the present study have been successful in identifying candidates for such follow-up studies, including a locus *(VAMP)* encoding membrane and secreted proteins that are required for intracellular survival of mycobacteria in macrophages, and KefB, a potassium transporter involved in control of early phagosomal pH and survival in the chronic phase of infection in mice [30].

A central aim of the present study was to test whether comparison of independent TSM datasets could provide insight into links between two complex biological phenotypes. Based on our understanding of mycobacterial interactions with macrophages, we had anticipated that we would observe an overlap between TSM datasets from screens involving mycobacterial survival and control of acidification. Unexpectedly, our results appeared to demonstrate that inability to inhibit phagosome acidification at 60 min post-infection did not confer attenuated survival and/or growth over the first 72 h of parasitism. However, a longer (6 d) infection with four individual mutants did demonstrate these strains to be defective for intracellular survival, but the phenotype was only just evident at 72 h and not overtly manifest until at least 96 h post-infection. Thus, while the TSM survival assay was successful in detecting those mutations that confer the most pronounced attenuation of survival, either it was not sensitive enough or it encompassed an insufficient infection time to detect the more subtle levels of attenuation observed in mutants that fail to control the pH of their phagosomes. It is also possible that mixed-infection experiments involving complex pools of mutants, such as the TSM assay, will fail to detect attenuation in certain mutants due to trans-complementation between strains. This may be particularly pertinent, given that many of the acid phagosome mutants have insertions in genes encoding secreted products that may be easily complemented by secretion from neighbouring bacteria carrying the wild-type gene. Our observations are in general agreement with the findings of Pethe et al. [28] who, using a selection procedure targeted at a later stage of phagosome maturation (interaction with dextran-containing lysosomes), isolated a set of mycobacterial mutants that were unable to block acidification and were also impaired in intracellular survival over a 9-d macrophage infection. However, none of the genes identified by Pethe et al. were found in our TSM dataset. One explanation for this lack of overlap may be that the different selection procedures used in the two studies have selected for different subsets of “phagosome maturation” mutants.

A broader comparison of the current data with published reports demonstrates a clear overlap between mutations that impair survival of BCG in macrophages and those that influence pathogenesis of *M. tuberculosis* in mice—for example, from Table 1, nine of 17 genes on which data are available in the study by Sassetti and Rubin [30] are also attenuated in the early stages of murine infection (using a cut-off of *p* < 0.05). This is consistent with previous reports of a concordance between macrophage survival and mouse pathogenesis, particularly during the initial stage of acute infection, when mycobacterial growth is controlled by innate immune mechanisms [32]. In contrast, very little overlap was observed between genes required for macrophage survival and those that are up-regulated during the early stages of macrophage infection [49]. Only two of the top 20 genes listed in Table 1 (the peptide transporter *dppC* and Rv1357c) were significantly up-regulated during macrophage infection, while expression of others, such as the *mce1* locus, was significantly depressed. Amongst the proteins encoded by genes implicated in control of acidification, PPE10 and two further PPE family members were significantly up-regulated during infection, as was the gene encoding the LppN lipoprotein. It is unlikely that there will be a simple linear relationship between essential function and changes in expression level, and linking these two datasets will require a deeper understanding of interactions between genes and their protein products at different time points during the course of infection.

## Materials and Methods

### Bacterial strains and growth conditions.

DNA plasmid construction was performed in *E. coli* DH5α using standard procedures. *Mycobacterium bovis* BCG (Pasteur) was cultured at 37 °C in Middlebrook 7H9 medium supplemented with 10% albumin/dextrose/catalase (ADC) and 0.05% Tween 80, or on Middlebrook 7H11 solid medium containing 10% oleic acid/albumin/dextrose/catalase (OADC) supplement. Hygromycin was included as appropriate at 50 μg/ml for BCG and 150 μg/ml for *E. coli*.

### Transposon mutagenesis.

The hygromycin-B phosphotransferase gene from *Streptomyces hygroscopicus* was cloned into the multiple cloning site of the EZ::TN pMOD transposon construction vector (Epicentre Biotechnologies, Madison, Wisconsin, United States) between the transposon Mosaic End sequences. The EZ::TN transposon containing the hygromycin resistance gene, EZ::TN*hyg,* was excised from the plasmid by PvuII restriction digest and gel-purified using QIAEX II (Qiagen, Valencia, California, United States). The transposome was generated by mixing EZ::TN*hyg* DNA with EZ::TN transposase (Epicentre) and glycerol according to manufacturer's instructions and incubated at room temperature for 30 min. Transposome mix (1 μl) was electroporated into 200 μl of competent *M. bovis* BCG, and transposition events were selected by plating on Middlebrook 7H11 containing hygromycin. Approximately 800 insertion clones were generated per electroporation, and 2,500 mutants were pooled by scraping into 7H9 broth with hygromycin and subcultured four times before macrophage infection. This pool of mutants did not provide complete coverage of the BCG genome, but we were reluctant to feed larger libraries into flow cytometry selection for fear of a bottleneck effect that would introduce stochastic variation into the selection output.

### Transposon screen by microarray.

We screened transposon libraries by microarray to reveal insertion sites and relative abundances of mutants using a modification of the techniques described by Badarinarayana [48]. Genomic DNA was prepared from pools of transposon mutants by standard procedures and restricted with BssHII to generate an average fragment size of approximately 900 bp. Oligonucleotides TA1 (5′-ACTACGCACGCGACGAGACGTAGCGTC-3′) and TA2 (5′-CGCGGACGCTACGTCCGTGTTGTCGGTCCTG-3′) were annealed to make a Y-shaped linker [48] with a BssHII-compatible 5′ overhang. Approximately 100 ng of digested genomic DNA was ligated to 100 pmol of Y-linker, and then 10 ng of ligated DNA was used as template for PCR amplification of transposon-flanking regions using the transposon-specific primer TA3 (5′-GCCTTCACCTTCCTGCACGACTTCGAGGT-3′) and the Y-linker-specific primer TA4 (5′-ACGCACGCGACGAGACGTAGC-3′) in the presence of 8% DMSO. Following an initial denaturation step for 2 min at 95 °C, the reaction was hot-started and cycled between 94.5 °C (30 s) and 72 °C (90 s) for 22 cycles. Amplification products less than 500 bp were gel purified and used in a second round of PCR amplification between the Y-linker-specific primer TA4 and a nested transposon-specific primer TA6 (3′-GTGTTCGAGGAGACCCCGCTGGATCTCTC-5′) to further enrich for transposon-flanking products and to incorporate Cy3 or Cy5-dCTP (Amersham, Little Chalfont, United Kingdom). The reaction mix included 20 μM Cy-dCTP, 180 μM dCTP, and 200 μM dGTP/dATP/dTTP and the cycling conditions were as before, but for 12 cycles. The labelled PCR products were cleaned up using a Qiagen MinElute kit, eluting in water.

The fluorescently labelled transposon insertion sites were hybridised to whole-genome microarrays, prepared by spotting the *M. tuberculosis* 70-mer oligonucleotide set (Qiagen and Operon [Huntsville, Alabama, United States]) onto Corning GAPS Coated Slides. Signal intensities of hybridisation were collected using Genepix Pro 3.0 and an Axon 4000B microarray scanner (Molecular Devices, Sunnyvale, California, United States).

### Macrophage infections.

J774 murine macrophage-like cells were grown in DMEM (Invitrogen, Carlsbad, California, United States) containing 10% heat-inactivated foetal bovine serum and 5 mM glutamine. For TSM screening of the BCG transposon mutants during infection, culture dishes containing 3 × 10^7^ macrophages were infected with 1.5 × 10^8^ bacteria from the BCG mutant library. Infections were synchronised by allowing the bacteria to adhere to host cells for 30 min at 4 °C before replacement of the inoculum with fresh complete DMEM and incubation at 37 °C and 5% CO_2_ for 1 h. The monolayer was washed three times with PBS to remove free bacteria and then either harvested for phagosome analysis or cultured in DMEM at 37 °C and 5% CO_2_ for analysis of intracellular survival and growth (Figure 1). We observed that approximately 35% of macrophages took up at least one bacterium, resulting in an expected internalisation of more than 4,200 bacteria of each mutant clone.

### Intracellular survival of transposon mutants.

To select for mutants with intracellular growth and/or survival defects, infected macrophages were cultured for 72 h and then the bacteria were harvested by washing the monolayer in PBS and lysing the macrophages by the addition of 7H9 medium containing Tween 80 and hygromycin**.** The resultant pool of transposon mutants was allowed to grow to late logarithmic phase and then used for a second round of macrophage infection and subsequently for a third selection through macrophages. The final output pool was grown to late logarithmic phase and processed for extraction of genomic DNA. The transposon-flanking regions were amplified, Cy-labelled, and co-hybridised with Cy-labelled genomic DNA from the input pool. The FR was calculated as output signal intensity divided by input signal intensity*.* Four replicates were performed and the entire experiment repeated.

Individual mutants isolated from the library were compared to wild-type BCG for survival and/or growth in J774 macrophages. Macrophages were seeded into 24-well plates at 2 × 10^5^ cells per well and infected with mycobacteria at a multiplicity of 1. The monolayers were washed and bacterial survival and growth assessed by plating of lysed monolayers on 7H11 and counting of colony-forming units.

### Flow cytometric analysis of mycobacterial phagosomes.

To detect mycobacterial phagosomes by flow cytometry, macrophages were infected with fluorescently labelled BCG. The bacteria were surface labelled by incubation with 1 μg/ml FITC in carbonate buffer (pH 9.2). During preliminary experiments, BCG were also labelled by heterologous expression of EGFP from plasmid pEGFPLux. No difference was observed in the localisation of FITC- and EGFP-labelled bacteria with respect to LysoTracker (unpublished data). After a 1-h infection, the cells were placed on ice, washed with ice-cold PBS, and scraped into homogenisation buffer (250 mM sucrose, 20 mM Hepes, 0.5 mM EGTA, 0.1% gelatine [pH 7.0]). The cells were lysed by multiple passage through a 22-gauge needle, and 20 mM ATP was added to break up the cytoskeleton. To remove nuclei, the homogenate was centrifuged at 900 rpm for 10 min and the supernatant retained and subjected to two further spins. The final post-nuclei supernatant was analysed on Becton Dickinson (Palo Alto, California, United States) FACSCalibur or FACSVantage flow cytometry systems. The frequency of EGFP- or FITC-positive events in different organelle populations, based on forward and side scatter, gave an indication of the position of the mycobacterial phagosomes. This was verified by sorting of different organelle populations and fixation for electron microscopy. Organelles were pelleted and fixed in 4% paraformaldehyde and 2.5% glutaraldehyde in PBS, followed by treating with 1% osmium tetroxide in sodium cacodylate buffer, rinsing with 1% tannic acid to highlight the phagosome membrane, dehydrating in an ethanol series, and embedding in Epon resin. Ultrathin 60-nm sections were further contrasted with uranyl acetate and lead citrate and viewed in a Philips CM100 transmission electron microscope.

To demonstrate the applicability of flow cytometry to differentiate phagosomes on the basis of the degree of acidification, we made a comparison of phagosomes isolated from macrophages infected with live and UV-killed BCG. BCG were irradiated in a Stratalinker UV crosslinker (Stratagene, La Jolla, California, United States) for 10 min. Preliminary experiments showed that this treatment rendered the bacteria unable to produce growth on 7H11 medium. As a marker for acidification of the phagosome we used the fluorescent acidotropic dye LysoTracker DND-99 (Invitrogen), which accumulates in acidified organelles and was included in media at 50 nM during 1-h infections of macrophages (see Figure 1). The threshold for determining LysoTracker positivity was determined by analysis of uninfected macrophages. The percentage of phagosomes that were positive for LysoTracker was determined by collecting data on at least 3,000 phagosomes.

### Flow cytometry selection of transposon mutants in acidified phagosomes.

Three culture plates of 3 × 10^7^ macrophages were infected with the FITC-labelled BCG transposon mutant library. After a 1-h infection in the presence of LysoTracker, the macrophages were harvested and organelles were prepared as above. The post-nuclei supernatant was analysed using the FACSVantage flow cytometer, and 2 × 10^6^ LysoTracker-positive mycobacterial phagosomes were sorted into 7H9 medium containing hygromycin. The sorted phagosomes were enriched with transposon mutants that were unable to arrest phagosome acidification. To further enrich for these mutants, the sorted bacteria were allowed to grow in 7H9 broth and were used for a second infection and flow cytometry selection, from which LysoTracker-positive and LysoTracker-negative phagosomes were collected. These pools of bacteria were grown in broth for extraction of genomic DNA.

The transposon insertion sites from the two genomic DNA samples were fluorescently labelled with different Cy-dyes as described above and co-hybridised to an *M. tuberculosis* microarray. Signal intensities of the transposon-directed labels were collected for the genomic DNA pools. The transposon labelling and hybridisations were repeated four times and included dye-swaps. The entire experiment was repeated.

### Confocal microscopy of phagosome acidification.

Macrophage infections with individual FITC-labelled transposon mutant strains and wild-type BCG were examined by fluorescence microscopy to assess acidification of phagosomes. Individual transposon mutant strains were identified and isolated from the library using insertion-specific PCR. Macrophages were seeded into chamber well slides overnight and then infected with BCG strains for 1 h in the presence of LysoTracker. The monolayers were washed three times and fixed in 2% paraformaldehyde in PBS before visualization using a Zeiss LSM510 confocal laser scanning microscope (Oberkochen, Germany).

### Data analysis.

Absent and poor-quality microarray spots were flagged using GenePix Pro 3.0 and median signal intensities calculated. For the intracellular survival experiment, data for input and output transposon pools were normalised to median signal intensities. Microarray data were analysed by significance analysis of microarrays [50]. This generates a *q* value, or FDR, for each data point. Survival of individual mutants in macrophages, and frequencies of co-localisation with LysoTracker obtained by flow cytometry and fluorescence microscopy, were analysed by Student's t-test. Predictions of prokaryote protein subcellular localization were made using PSORTb v.2.0 software (http://www.psort.org/psortb/).

## Supporting Information

Figure S1 Reproducibility of the TSM Labelling and Genome-Wide Distribution of Transposon Insertions (A) Reproducibility of the TSM labelling and hybridisation. Hybridisation signal intensities were compared from two independent labelling and hybridisation operations on a 2,500-clone EZ::TN*hyg* insertion library. A strong correlation was observed (*r^2^* = 0.9289).
(B) Genome-wide distribution of transposon insertions in a pool of 2,500 *M. bovis* BCG EZ::TNhyg mutants revealed by PCR-based TSM using an *M. tuberculosis* H37Rv microarray. The horizontal bar represents the genome of H37Rv with mutagenised genes represented as vertical black lines. Three of the BCG deletion regions are indicated by arrows along with a region of defective oligonucleotide probes that do not efficiently hybridise target DNAs.
(848 KB PDF)

Figure S2 Mutants with Defects in the Inhibition of Phagosome pH Are no more Likely to Have Reduced Intracellular Fitness than Those in a Random Mutant Library (A) Distribution of intracellular fitness values (FR) in the acid phagosome mutants. Mean, 0.146; 95% confidence interval = 0.223.
(B) Distribution of intracellular fitness values in the whole transposon library. Mean, 0.024; 95% confidence interval = 0.042.
(394 KB PDF)

Table S1 Ez::TN*hyg* Insertion Sites (21 KB XLS)

Table S2 Fitness Ratios of All Mutants During Growth in Macrophage Culture (276 KB XLS)

Table S3 The 100 Mutants Most Attenuated During Growth in Macrophage Culture (28 KB XLS)

Table S4 Mutants Enriched in Acidic Phagosomes (22 KB XLS)

## Abbreviations

BCG: bacille Calmette-Guérin
DIM: phthiocerol dimycocerosate
FDR: false discovery rate
FR: fitness ratio
SD: standard deviation
TGN: trans-Golgi network
TSM: transposon screen by microarray
VAMP: virulence-associated membrane protein
V-ATPase: vacuolar proton ATPase
