# Supplementary material for: Mycobacterial Mutants with Defective Control of Phagosomal Acidification
Source: PLoS Pathog. 2005 Nov 25;1(3):e33. doi: 10.1371/journal.ppat.0010033 (PMC1291353; doi:10.1371/journal.ppat.0010033)
Supplement: Figure S1 — (A) Reproducibility of the TSM labelling and hybridisation. Hybridisation signal intensities were compared from two independent labelling and hybridisation operations on a 2,500-clone EZ::TNhyg insertion library. A strong correlation was observed (r2 = 0.9289). (B) Genome-wide distribution of transposon insertions in a pool of 2,500 M. bovis BCG EZ::TNhyg mutants revealed by PCR-based TSM using an M. tuberculosis H37Rv microarray. The horizontal bar represents the genome of H37Rv with mutagenised genes represented as vertical black lines. Three of the BCG deletion regions are indicated by arrows along with a region of defective oligonucleotide probes that do not efficiently hybridise target DNAs. (848 KB PDF) [file ppat.0010033.sg001.pdf]

Supplementary Fig1A

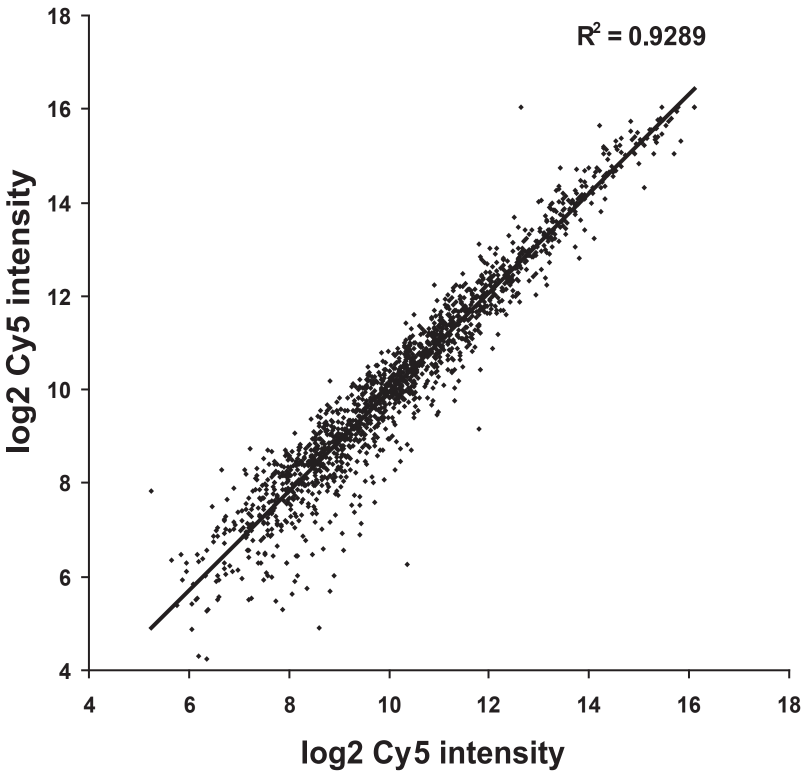

Supplementary Fig1B

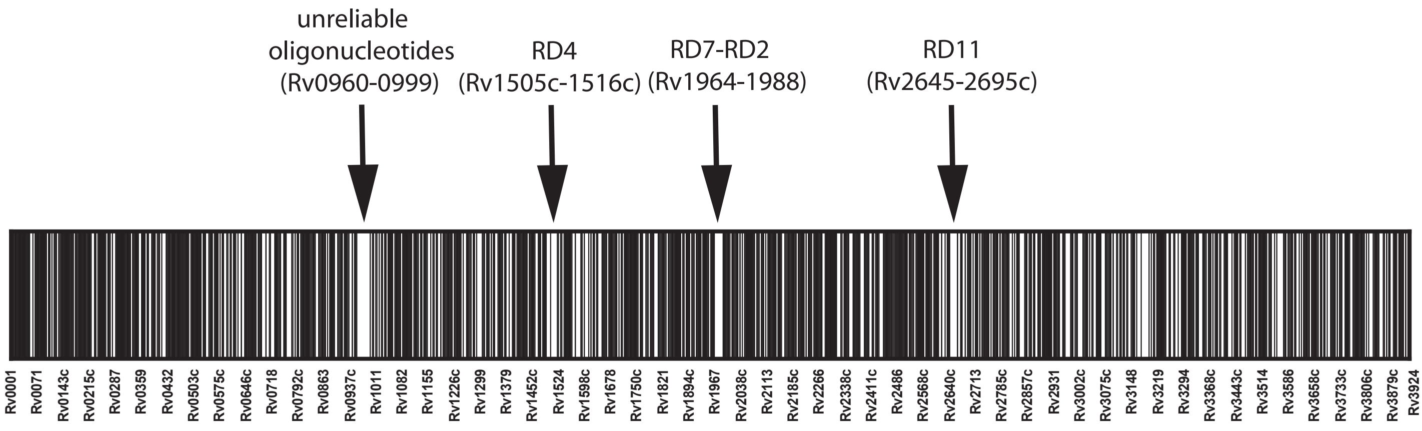

Supplementary Figure 1A. Reproducibility of the TSM labelling and hybridisation. Hybridisation signal intensities were compared from two independent labelling and hybridisation operations on a 2500-clone EZ::TNhyg insertion library. A strong correlation was observed (R2=0.9289).

Supplementary Figure 1B. Genome-wide distribution of transposon insertions in a pool of 2500 M. bovis BCG EZ::TNhyg mutants revealed by PCR-based TSM using an M. tuberculosis H37Rv microarray. The horizontal bar represents the genome of H37Rv with mutagenised genes represented as vertical black lines. Three of the BCG deletion regions are arrowed along with a region of defective oligonucleotide probes which do not efficiently hybridise target DNAs.
