# Supplementary material for: Mycobacterial Mutants with Defective Control of Phagosomal Acidification
Source: PLoS Pathog. 2005 Nov 25;1(3):e33. doi: 10.1371/journal.ppat.0010033 (PMC1291353; doi:10.1371/journal.ppat.0010033)

**Figure S2. Mutants with defects in the inhibition of phagosome pH are no more probable to have reduced intracellular fitness than those in a random mutant library. (A) Distribution of intracellular fitness values (fitness ratio, FR) in the acid phagosome mutants. Mean =0.146, 95% confidence interval = ±0.223 (B) Distribution of intracellular fitness values in the whole transposon library. Mean 0.024, 95% confidence interval = ±0.042.**

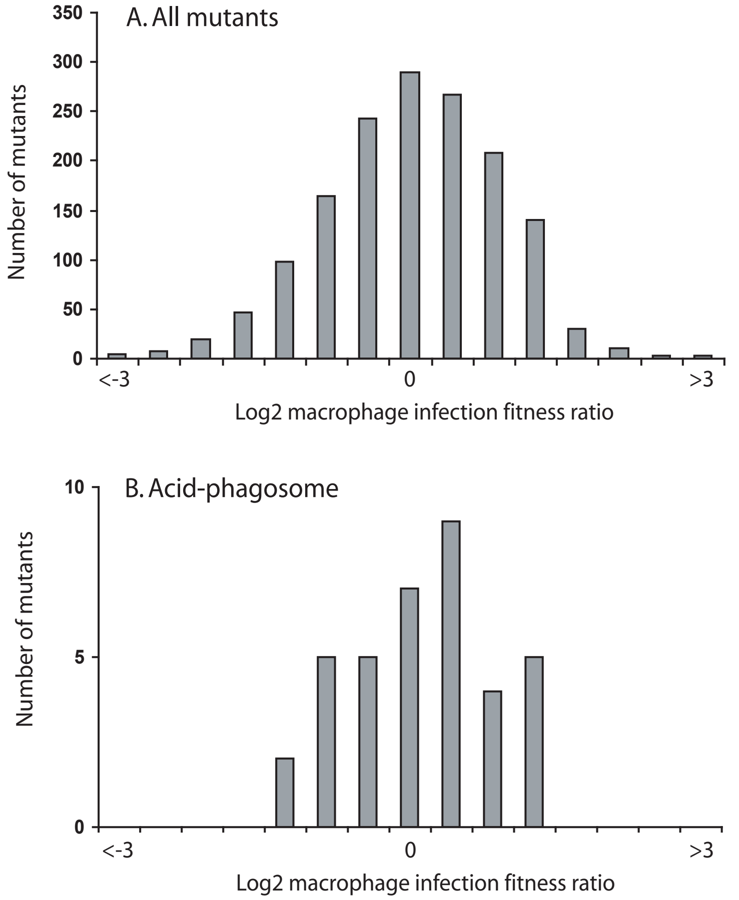

Supplement: Figure S2 — (A) Distribution of intracellular fitness values (FR) in the acid phagosome mutants. Mean, 0.146; 95% confidence interval = 0.223. (B) Distribution of intracellular fitness values in the whole transposon library. Mean, 0.024; 95% confidence interval = 0.042. (394 KB PDF) [file ppat.0010033.sg002.pdf]
